# Observations *on the* Effects *of* Mercury *in Its* Native State, *and on Its* Abuse *in Certain* Diseases

**Published:** 1813-03

**Authors:** John W. Francis

**Affiliations:** New York


*For the Medical and Physical Journal.*

OBSERVATIONS *on the* EFFECTS *of* MERCURY *in its* NATIVE STATE, *and on its* ABUSE *in certain* DISEASES. *By* JOHN W. FRANCIS, A.M. M.D. &c. New York.

AMONG the earliest authors who have bestowed attention on this subject, were George Agricola, who noticed the destructive effects of mercury on the human constitution, in his work on Minerals, and Johannes Francis Fernelius, a contemporary writer, in his Treatise on Lues Venerea, whose respective productions appeared in the early part of the sixteenth century. The latter author makes known known the case of a goldsmith, who, upon imprudently exposing himself to the vapor of quicksilver, "became stupid, lethargic, and quite dumb."* Similar to this is the declaration of Boyle, who observes that death has been occasioned from the same cause.† Besides these authors, may be mentioned the Jesuit Acosta,‡ Fabricius Hildanus,§ and Bernadin Ramazzini.‖ Among the consequences attendant on those who work in mercury, or are exposed to its fumes, Hoffman states palsies, apoplexies, epilepsies, hectic fever, &c.¶ Paulus Zaccheus, in his legal medicine, enters at length into the consideration of the effects of mercury in its native state; and not only declares his own opinion of their pernicious nature, but adduces arguments in confirmation, from some of the early Arabian writers, and enumerates the diseases caused by the fumes of this metal; he adds, *the same Avicenna declares.*** "M. Jussiau," says Dr. Alley, "in a paper on the working of the quicksilver mines of Almaden, in Spain,

* Fernelius de Lue Vener. p. 590. Aston Mat. Med. vol. i. p. 80.

† Works, vol. iii. p. 330, fol. ed. 1744.

‡ Discourse of Quicksiluer, of the Mynes and Worke, and what is requisite for that subject. Purchas his Pilgrimes, vol. iii. p. 947-8.

§ Observat. et Curat. Chirurg. cent. v.

‖ De Morbis Artific. p. 491.

¶ Dissert. Metallurg. morbif. Rational. Systemat. vol. ii. ed. 1729.

** Ut idem Avicenna testatur. Quest. Medico-legal. lib. xi. p. 212, ed. 1688.

published published in 1719, mentions the appearance of pustules over the whole body as a consequence of a contact with the mineral, even in its metallic state, together with tumors of the parotids, aphthæ, and salivations."*

To the authors already mentioned might be added many others of more modern date, whose declarations are in unison with what has been just advanced; but a general reference to their works must suffice. See the respective publications of Dr. Mead,† M. Sauvages,‡ Dr. Brown,§ and the Abbé Raynal.||

The writer cannot, however, forbear to introduce on this subject, the remarks of Professor Gmelin, of Goettingen, as contained in his Apparatus Medicaminum, a recent performance of the German press. They are the result of extensive examination, and possess additional claims to attention from the authorities he has adduced in support of their correctness. "Neque dubius est mercurii in nervos effectus, funestus omnino, ut ea certe forma veneni nomine dignissimus sit, si halitus specie per nares et os corpus ingrediatur, ita ut non tremores modo artuum experiantur, v. gr. metallici, qui in fodinis, in quibus mercurius virgineus occurrit, operantur: chirurgi qui in mercurio per frictionem adplicando desudant, et ii qui, e. g. in officinis venetis mercurii cum stanno mistura vitri superficiem illinunt, sed alii quoque artifices, qui sergentibus ex igne mercurii vaporibus sese exponunt potissimum aurifabri, qui in deaurandis ignis auxilio aliis metallis versantur, tum asthmati, tum paralysi artuum, et apoplexiæ præsertim valde sint obnoxii, neque rara adeo sint exempla hominum, ab inspiratis exitiosis his halitibus mortem repentinam obeuntium."¶

The preceding opinion relative to the destructive effects resulting from the fumes of mercury, though grounded upon such weighty testimony, has been rejected by a late and eminent writer, the Baron de Humboldt; who attributes the pernicious consequences which ensue from the working of mines of that metal, to the excessive labor the individuals engaged in this employment undergo. "From five to six thousand persons," says he, "are employed in the amalgamation of the minerals, or the preparatory labor. A great number of these individuals pass their lives in walking

---

* Observat. on Hydrargyria, p. 4.

† Medical Works, vol. i. p. 106.

‡ Nosolog. Method. vol. i. p. 558, ed. 1768.

§ Travels, Harris's Collect, vol. ii. ut antea.

|| East and West Indies, vol. iii. p. 144.

¶ Apparat. Medicam. *(regnum minerale complectens.)* vol. ii. p. 22.

 barefooted barefooted over heaps of brayed metal, moistened and mixed with muriate of soda, sulphate of iron, and oxide of mercury, by the contact of atmosphere and the solar rays. It is a remarkable phenomenon to see *these men enjoy the most perfect health*."* He also declares, that a part of the inhabitants, at Guanaxuato, drink the very water in which the amalgamation has been purified, without feeling any injury from it.

There is no doubt that certain circumstances are required to induce the morbid operation of mercury, even in this state, but these circumstances are of the very nature of those observed by the Baron himself. It is requisite that the metal undergo a minute subdivision, in order to exercise a pernicious tendency upon the body; a principle declared by Hoffman, and ably illustrated by Dr. Mitchill, the ingenious and learned professor of natural history in the university of New York.† In corroboration of this remark, may be stated the very recent occurrence which took place on board two vessels, the Triumph and Phipps, in which, from the fumes of quicksilver, an alarming illness broke out among the crews, all of whom were more or less salivated.‡ Reasoning from analogy, a like conclusion may be drawn from the effects of other metallic ores.§ The facts, too, observed by Robert Boyle,|| which are diametrically opposed to the latter declaration of Humboldt, might be here added, had not this part of the subject already been extended to too great a length. It remains with the reader to decide whether the evidence advanced affords a sufficient refutation of the observations of the Baron.

The materia medica contains not an article in the employment of which there are more circumstances deserving consideration, than those connected with or attendant upon the use of mercury. This remark with nearly equal force applies to all the various forms in which this powerful remedy is, at the present day, administered; and he who ventures to depend upon the successful operation of this medicine, without a due regard to the age, sex, and particular constitution, of the patient; the diagnostic symptoms and

---

* Political Essay on the Kingdom of New Spain, vol. i. p. 127. Lond. 1810.

† MS. Notes of Mitchill's Lectures, (penes me.)

‡ Edin. Med. and Surg. Journal, vol. vi. p. 513.

§ See Franklin's Letter to Vaughan, Works, vol. iii. p. 398. Phil. 1808.

|| Boyle on the strange Subtility of Effluvium, Works, fol. ed. vol. iii. p. 317.

stage stage of the disease; the previous treatment that may have been pursued; climate, and temperature; will not only subject himself to frequent disappointments, and hazard the safety of his patient, but most deservedly become the object of severe crimination. The whole history of mercury, in the numerous and complicated diseases in which it is employed, justifies the correctness of this assertion; and, would it not far exceed the limits proper for this place, abundant evidence, derived from unquestionable sources, might be here detailed.

Mercury, when taken into the system in considerable quantity, either through the mouth or by absorption from the surface, acts with peculiar force upon the stomach, inducing indigestion, loss of appetite, nausea, and vomiting; the functions of the chylopoietic viscera become greatly disturbed, especially those of the liver; and, from its very powerful effects upon the digestive organs, the whole intestinal canal soon participates in the disorder. It exerts a no less powerful influence upon all the secretory and excretory vessels of the body, as in those of the mouth and fauces, trachea, bronchiæ, and lungs; and, not unlike the preparations of antimony, paralyses the action of the minute and distant vessels, especially those of the lymphatics. From this most general operation, a languor of the whole system is produced, the constitution at large becomes affected, and symptoms highly indicative of general derangement and nervous irritability ensue. These effects, however, are much more readily induced in particular individuals or habits of body than in others; and, as remarked before, an intimate relation exists between the operation of this medicine, and the age, sex, constitution of the patient, temperature, &c.

A small dose of calomel, given in the infantile state, has been followed by convulsions, and sanguineous discharges from the alimentary canal.* In some instances, the same preparation of mercury, in small doses, has excited a preternatural action of the blood-vessels, and hæmorrhage from the lungs; thus predisposing to consumption, and ending in that disease; and, in females of delicate habit, violent menorrhagia. In certain constitutions it has given rise to symptoms characteristic of dysentery, which have been succeeded by a colliquative and fatal diarrhœa. Examples, too, are not wanting, of a most profuse salivation, followed by extreme weakness and great prostration of strength, being attendant upon the exhibition of but a few grains of this

* MS. Notes of Hosack's Lectures on the Mat. Med.

 medicine, medicine. Indeed, Dr. Ferriar relates the case of a maniac, to whom even half a grain of calomel proved a full dose.* Its peculiar effects upon the minute and distant vessels of the absorbent system, depending either upon the excitement induced, or upon the debility consequent thereon, are fully apparent when the kinds of diseases which it occasions are considered; such as the several forms of dropsy, hydrocephalus, hydrothorax, and anasarca, and various affections of the lymphatic and glandular system; and, when this principle of its action is considered in its full force, little doubt need be entertained, that, of the numerous causes which have been assigned for the preternatural decay of teeth, mercury may be justly reckoned a principal one.†

To these more ordinary ill effects attendant upon the frequent and indiscriminate use of mercury, may be added the numerous disorders which it induces of the nervous system, and of the intellectual functions; as, among the former, tremors, convulsions, epilepsy, palsy, &c. A remarkable instance of this last disease is related by Professor Hosack, as having occurred in a child of this city, who, from the abuse of this remedy, was altogether deprived of the use of its limbs.‡ Of the derangements of the intellectual system, might be mentioned the various affections of the mind, from a state of vigilance to perfect mania; and not uncommonly an impaired state or total deprivation of one or more of the senses, as loss of sight, loss of memory, loss of voice, and deafness. In the case just noticed, so powerful were the effects of mercury on the memory, that the child, although able to read previous to its employment, was compelled to relearn the letters of the alphabet. But these facts, though of the greatest value, possess little claim to novelty. Many of them were noticed by practical observers, soon after the introduction of this mineral as an article of medicine; and, at the present day, as the importance of the subject has become more manifest, it has deservedly acquired a proportionably greater degree of attention. But, without further enlarging, in this general manner, the catalogue of ills which originate from the abuse of mercury, it will probably be attended with more practical advantage to consider the present fashionable employment of this remedy in the treatment of particular diseases; and, for reasons which must appear obvious,

* Med. Hist. and Reflect. vol. iii. p. 256. See also Pearson on the Effects of various Articles in the Cure of the Lues Venerea, p. xix.

† See also Fox on Diseases of the Teeth, p. 108.

‡ MS. Notes on Lectures on the Mat. Med.

these remarks are necessarily confined more especially to the practice pursued by the physicians of our own country. And, first, of some of the diseases belonging to the class denominated febrile.

To the bold and vigorous practice adopted by the celebrated Dr. Chisholm, in the cure of the malignant pestilential or yellow fever which prevailed at Grenada, and at other of the West-India islands, in 1793, may be attributed the present general employment of mercury in the treatment of all the various forms of intermittent, remittent, and typhus fever in the United States. This remedy, it is well known, was long before that time made use of, and often to a considerable extent, in the treatment of the febrile diseases of hot climates, and also in some diseases of a like nature which prevailed in this country. Dr. Warren, in his treatise, published in 1740, animadverts on the practice of giving calomel in inflammatory fevers.* Dr. Gilchrist, shortly after that period, expresses a high opinion of the superior virtues of mercury in the low state of fever, during the existence of inflammation.† Dr. Wright employed it in the acute diseases of Jamaica, in 1764; and the same remedy was resorted to by Dr. Chisholm himself, in the cure of the yellow remitting fever which prevailed at Grenada in 1786.‡ In our own country, during the existence of the sore-throat distemper, about the year 1740, (a disease in some respects precisely of the nature of the malignant yellow fever, according to Dr. Chisholm,) frequent recourse was had to this medicine, and in no small quantities, by Dr. Ogden, of Long Island.||

But, though the *remedy* introduced in Grenada in 1793 was by no means new in the treatment of other disorders, to which the malignant pestilential fever, in many particulars, bore a strong resemblance, yet to Dr. Chisholm exclusively belongs the credit of being the first who adopted the novel practice of exhibiting mercury to the great extent it is now employed in this disease; a practice to which he was led from the ineffectual result of every other remedy, and from the appearances he perceived in the two first bodies he opened. In some cases, mercury, in the form of calomel, was given to an almost incredible extent; frequently to the amount of four hundred grains; and during the existence of the same epidemic in subsequent years, sometimes no less

* Treatise on the Malignant Fever of Barbadoes.

† Edin. Essays and Observ. Physical and Literary, vol. iii. p. 498.

‡ Duncan's Med. Comment. Dec. 1. vol. i. and viii.

|| Mitchill and Miller's Med. Rep. vol. v. p. 98.

than than eight hundred, and at other times upwards of one thousand, grains were employed. But the success of this practice, says Dr. Chisholm, "justified my temerity;" and his future experience and observation produced the fullest conviction of its efficacy. An extraordinary instance of the effects of mercury, in the treatment of the *yellow remitting fever*, may be here noticed: before any very material change took place in the state of the patient, "he had taken," says Dr. C. "sixty-four grains of calomel by the mouth; thirty-four drachms, or two thousand and forty grains, were administered by clyster; and sixteen ounces of the strongest mercurial ointment, or about three thousand six hundred grains of triturated mercury, were carefully rubbed into his arms and thighs; in all, five thousand seven hundred and four grains, in the course of five days. His recovery was astonishingly rapid after the favorable change was effected."*

Every consideration conspires to induce the fullest belief, that the mercurial practice was adopted by Dr. Chisholm from the most laudable motives; and the Essay which he has offered to the world, while it affords a luminous and faithful exhibition of the ravages committed by a dreadful pestilence, and of the means employed to arrest its progress, presents the most unquestionable evidence of superior intellect, united to a heart equally alive to every ennobling sentiment, and zealous for the best interests of human kind.

This digression on the practice of Dr. Chisholm seemed proper and necessary; as, upon the appearance of the yellow fever, in the same year, in the city of Philadelphia, though depletion by the lancet constituted a chief part of the method of cure, the mercurial plan of treatment was also adopted. During the prevalence of the same epidemic in 1794, in Newhaven, Philadelphia, and Baltimore, the application of this remedy became more general, particularly in the last-mentioned city; not, however, without calling forth the animadversions of some, who contended that the principles upon which it was administered were erroneous, and the practice itself pregnant with mischief. But the nature of the present performance forbids farther minuteness. Let it suffice to observe, that, upon the re-appearance of the yellow fever in the various parts of the United States, at every subsequent period, many eminent practitioners had immediate recourse to the mercurial method, which was pursued until salivation was excited; a

* Essay on the Malignant Pestilential Fever, vol. i. p. 476.

practice upon which they placed their chief reliance; while, on the other hand, no inconsiderable number of medical men, of great professional reputation, adopted it but partially, and with great reluctance, or indeed rejected it altogether.*

As to the *success* attendant upon the mercurial practice in the yellow fever of this country, whatever it might have been in comparison with that resulting from any other method of treatment, it is abundantly evident, from the mortality which occurred, that in this respect here, as well as in the malignant fever of the West Indies, much room was left for improvement.

The utility of the free employment of mercury in the treatment of the yellow fever of the United States, may be well doubted; chiefly for the following reasons:

First. The climate of this country is singularly unfavorable to the salutary operation of this medicine. The frequent and uncertain transitions of the weather from heat to cold, from dryness to humidity, while they are often the cause of sudden and violent diseases, which generally assume the inflammatory character, they give the body a certain morbid predisposition, that renders it unable to withstand the influence of a mercurial course, which, on the native of a more southern and temperate atmosphere, would at least prove not injurious, and probably successful. However numerous and contradictory the reasons assigned by pathologists may be, the fact itself cannot be questioned; though it does not seem to have been duly regarded by those who were desirous of establishing in this country the practice of the West-India physicians.

Secondly. The morbid appearances, as they are exhibited upon the dissection of individuals who have died of the

---

* See Professor Rush's several histories of the bilious yellow fever in Philadelphia, in Med. Inq. and Obser. vol. iii. and iv. Drysdale's account of the yellow fever of Baltimore in 1794, in Coxe's Medical Museum, vol. i. Monsons on the yellow fever of Newhaven in 1794, in Webster's Collect. of Papers on the Bilious Fever. Smith's letters to Dr. Buel on the yellow fever of New York, in ditto. Bayley on the epidemic fever of New York, in 1795. Hosack on the yellow fever of New York, in 1798 and 1799, in Currie's Sketch of the Yellow Fever of Philadelphia, in 1799. Rand on the epidemic of Boston in 1798, in the Med. Repos. vol. ii. Caldwell on the yellow fever of Philadelphia, in 1803 and 1805. Currie's respective publications on the synochus icteroides. Wheaton on the yellow fever of Providence, R. I. in Med. Repos. vol. x. and other papers by different American writers, in the same Journal.

yellow fever in different parts of the United States, during its prevalence in different years, furnish another argument against the extensive employment of mercury in this disease. As it is universally admitted that this remedy is a powerful deobstruent, and indicated in all cases of local congestion, particularly of the glandular system; from a supposition that this part of the body was especially effected, implicit recourse was had to mercury. But, while the liver appears to be the most diseased organ in those who die of yellow fever in the West Indies, as Dr. Chisholm and others have declared,* this important viscus seems to be in a remarkable degree exempt from derangement, in those who have died of the same disease in this country. Such was found to be the case, in the fever of Philadelphia in 1793, by Doctors Physic and Cathrall; † in those who died of the yellow fever in Newhaven in 1794, as remarked by Dr. Monson; ‡ in the fever of the same year in Philadelphia, and in that of 1798, according to Dr. Rush; § and again from the dissections made in the same city in the epidemic of 1805, as made known by Dr. Caldwell; ‖ and by Dr. Lowber, in the Pennsylvania hospital.** Exceptions, however, occurred in the yellow fever of Boston in 1798. ††

As dissections thus show that congestion or infarction of the liver is of rare occurrence in the yellow fever of this country, mercury, therefore, is seldom serviceable on account of its specific action on the hepatic system; and the impropriety of inducing salivation as a general remedy to remove glandular obstructions, which rarely take place, upon the principle that they constitute the proximate cause of the disease, is too striking to be overlooked. But there are other circumstances which more directly contra-indicate the indiscriminate use of this remedy in yellow fever. The very nature and seat of the disease, as they are characterized by symptoms which uniformly accompany it, and by numerous dissections, establish the fact, that the brain and nervous system are primarily affected; that great disorganisation constantly occurs in the digestive organs, the stomach

* Essay on the Malig. Pest. Fever, vol. i. p. 351.

† Rush's Med. Inq. and Observ. vol. iii. p. 174. Cathrall's Sketch of the Synochus Maligna, p. 70.

‡ Webster's Collection on Bilious Fevers, p. 189.

§ Med. Inq. and Observ. vol. iii. p. 371. vol. iv. p. 77.

‖ Essay on the Pestilential or Yellow Fever of Philad. in 1805, p. 100.

** Phil Med. Museum, vol. v. p. 16.

†† Rand and Warren's Account, Med. Rep. vol. ii. p. 240.

and and duodenum commonly exhibiting marks of high inflammation, and approaching sphacelus, and at other times appearances similar to those which ensue from the action of the most narcotic poisons;* and that not unfrequently the same morbid effects extend throughout the whole intestinal canal. Besides this, let the reciprocal action of the brain and of the digestive organs on each other be considered, and the intimate connection which exists between local disease and the disorders of the whole system, as the phenomena resulting from them are particularly evident in yellow fever, and the position before advanced receives strong additional support.

It is scarcely necessary to remark, that mercury seldom fails to weaken the constitution, and always to impair the digestive functions of those even who in these respects are in a sound state. How then is it practicable, by the use of calomel, to diminish those acute pains in the gastric region, or to allay that extreme irritability of the stomach, which are the concomitants of yellow fever? The suggestions of reason are confirmed by experience. These symptoms, instead of being removed, are greatly aggravated, and, while the acute gastric affection becomes still more violent, an excessive vomiting is induced, which, in many cases, terminates but with the life of the patient. The observations of the venerable Dr. S. Bard, and of Dr. Hosack of New York, of Dr. Currie of Philadelphia, of Dr. Chatard of Baltimore, and of Dr. Wheaton of Providence, during the prevalence of this disease in those respective places, are directly in support of this fact.

But it is declared that the success of the mercurial practice depends upon salivation, and the recovery of the patient has been pronounced to be certain whenever this effect of mercury was perceived to take place. As, however, inflammatory symptoms in numerous instances constitute the first stage of yellow fever, when the peculiar operation of mercury during their predominance is considered, and the impracticability of exciting salivation, it must be acknowledged that this remedy is but ill calculated to mitigate the violence, or to arrest the progress, of the disease. Equally apparent is it, that in the more advanced stage of the complaint, when the more formidable and alarming symptoms have already taken place, a reliance upon mercury will be attended with disappointment. The great prostration of strength under which the patient now labors, forbids recourse to a medicine

* See also Observations on that form of Pestilence called Yellow Fever, by Edward Miller, M.D. Med. Rep. vol. ii. p. 379.

the action of which is so powerfully debilitating; the rapid termination of the disease sets at defiance the slow and uncertain accomplishment of salivation.

There is yet another argument which remains to be considered, which cannot but still more strongly confirm the doubts concerning the propriety of depending upon the action of the salivary glands as a remedy for yellow fever. Salivation, even in those cases in which it is produced, does not invariably secure the safety of the patient. Two instances in support of this fact fell under the observation of Dr. Hosack, in which, though salivation had been brought on by mercury, it did not prevent the fatal termination of the disease, nor was its beneficial effects manifested by any alleviation of its symptoms. Several other cases of a similar kind occurred to Dr. J. C. Osborn, of this city, and many others came to his knowledge while he practised physic during the prevalence of the yellow fever in North Carolina in 1799, which were attended with the same result.

Having thus examined, with as much brevity as the importance of the subject admits, the claims which mercury is said to possess in the treatment of the yellow fever of the United States, and attempted to show that they are not only altogether unfounded, but that the remedy itself, employed in the manner recommended, is highly inimical to the constitution, perhaps it is incumbent on the writer to state a preferable practice. This, however, formed no part of the plan he originally proposed, neither can he be considered competent to so responsible an undertaking. Yet, if it be allowed to offer his ideas on this subject, he does not hesitate to declare, that what may be denominated the *sudorific* plan, appears to be by far the most successful. After the necessary evacuations from the stomach and bowels, and in some instances from the blood-vessels, by the lancet, the administration of those remedies which relax the surface of the body, and promote the cuticular discharges, are particularly indicated; these kept up for some time, are found to be the most efficient means in the cure of yellow fever.

The *abuse* of mercury, as before remarked, is not confined to the treatment of the yellow fever. General recourse is now had to the same remedy, for the cure of all the various forms of *intermittent* and *remittent fever* which prevail in different parts of our country, and for every variety or type of fever, from the *purest inflammatory* to the *lowest grade of typhus*. Upon whatever principles of pathology the practice is attempted to be maintained, the fact is indubitable, and too singular to pass over without animadversion. The operation of the several functions of the body, and the derangements ments of particular organs, are altogether disregarded, (though the phenomena of morbid action in many cases originate from local affections;) and, upon the conjectural idea of *general disease*, one remedy, modified according to the peculiar notion of each individual, is alone indicated.

But there are other reasons assigned in justification of the mercurial practice in these diseases. The primary cause of intermitting and remitting fevers, it is said, depends upon some derangement in the secretory vessels of the liver; these, it is contended, must be unlocked in order to effect a cure, and mercury is the key to the hepatic system. Hence it is necessary to saturate the body with mercury. As to the febrile diseases characterised by another train of symptoms, in which all the marks of pure synochal fever are present, on the principle that they arise from a general fulness and preternatural action of the vessels, it is essential for the cure to diminish the former and allay the latter, and mercury is now employed to attain both these objects. With regard to typhus, as it is distinguished by a disturbed state of the sensorial functions and great prostration of strength, these symptoms are to be removed by restoring the general excitement, and giving proper tone to the system. Mercury is now said to possess, in an eminent degree, the property of restoring and equalizing the excitement of the whole system; its tonic power is its "primary operation," and in its primary operation it "communicates general vigor."* Thus it is that a single remedy possesses a "universal fitness," and is admirably calculated for all the various kinds of febrile disorders! The pernicious consequences resulting from such practice are next to proverbial.

In the treatment of the diseases universally acknowledged to arise from *specific contagion*, mercury has at length become an active constituent in almost all the various articles employed for that purpose; and there is every reason to believe that the evils resulting from such practice have greatly counterbalanced the advantages. But the consideration of one only of those forms belonging to this class can be here attempted. Notwithstanding all that has been written, so ill defined are the diagnostic signs of *lues venerea*, whether it assumes the form of a local affection, or operates more immediately on the whole system; so various and complicated are its symptoms in its different stages, and so uncertain its course and termination, that there are few complaints which require a more intimate knowledge of their nature in

* Murray's System of Mat. Med. vol. i. p. 213.

order to effect a radical cure; and it may well be questioned, whether the mal-administration of the remedy has not produced as destructive consequences as the disease itself. The practical observer will recollect how numerous are the diseases arising from causes essentially different from that which gives origin to the venereal, yet, nevertheless, in their characters bear an exact resemblance to it; that the venereal not unfrequently exists in combination with other disorders; and that, from errors in the mode of treatment, not only its nature may become altogether changed, but diseases equally formidable be brought on.

The existence of diseases of the first kind, "wearing the livery" of lues venerea, did not escape the acute penetration of Mr. Hunter; and the subsequent labors of Mr. Abernethy have more fully disclosed their perplexing nature. But, as there are no discriminating marks by which they may be distinguished, there is yet no general rule of practice established. The effects of mercury upon them are various. According to Mr. Abernethy, they are sometimes cured by it, sometimes they are only checked, and at other times aggravated.* This, however, is certain, that mercury may be misapplied in the treatment of these anomalous cases; and the practical caution of Mr. Hunter is not to be forgotten; that "it is nearly as dangerous, in many constitutions, to give mercury where the disease is not venereal, as to omit it in those which are." † In the diseases of the second kind, it is equally well known, after the removal of the specific infection for which recourse was had to mercury, that the other symptoms under which the patient may labor are so deceptive, as to be enumerated among the secondary effects of the venereal, and, instead of yielding to the power of the same remedy, become more alarming, and, if its use be persisted in, terminate fatally. And, as far as relates to the cure of this disease, those most conversant with it have furnished sufficient proofs of the pernicious consequences attendant upon errors in its treatment. ‡

Of these errors, the common, and probably the most destructive, is the inducing of profuse salivation, which is

* Surgical Observations, Part II. on Diseases resembling Syphilis.

† Hunter, ut antea, p. 381.

‡ See the respective publications of Hunter, Bell, Swediaur, and Howard, on the Venereal. Pearson on the effect of various articles in the cure of Lues Venerea. Alley's Observ. on Hydrargyria. M'Mullin on the Erythema Mercuriale, Ed. Med. and Surg. Journal, vol. ii. p. 25. Willan on the Diseases of the Skin; and Mathias on the Mercurial Disease.

generally generally brought on by throwing into the system large quantities of mercury. To do this in the shortest possible time is the immediate object, and calomel pills, or some other form of mercury, is taken internally, and mercurial unguents or frictions employed. The evident result of such practice cannot fail to be injurious to the constitution. When this discharge is thus excited, it often continues until a total exhaustion of the strength of the patient. In many cases, where it has been thought to have removed the disease, it has proved to be only a temporary cessation; and in other instances, it has converted a comparatively mild disorder into one infinitely more dangerous.

As the venereal arises from the introduction of a specific morbid matter into the system, so the peculiar action of this matter constitutes the disease. The particular manner in which it produces the various changes from a healthy to a diseased state, though too subtle to be observed by the senses, appears to be sufficiently well understood, from the effects which take place. They, indeed, are numerous, but probably depend chiefly upon an altered and impure condition of the fluids, from which sources all parts of the body may be affected. The idea of a ferment, therefore, or, rather, of an *assimilating process*, is most consonant to the phenomena which accompany the operation of the specific matter of lues venerea. The poison of disease may be taken up by the absorbents, and a local affection be induced; this soon becomes general, according to the virulence of the matter, and the susceptibility of the constitution.

If these principles be correct, it follows, that in the cure of venereal, such remedies are to be employed as operate more directly in promoting the action of all the secretory vessels of the body, and especially those of the surface; because, as before stated, by this action the morbid process which has taken place will be arrested, and the assimilated matter carried out of the body. Equally opposed, therefore, to the opinion declared by Mr. Bell, "that no advantage is derived from any increase that may be made to any of the secretions,"* and to that of Mr. Howard, who is the advocate for profuse salivation, even in the mildest form of the disease, and who contends that the greater the discharge the more certain the cure;† the truth lies between them, and the most certain and effectual practice depends upon an increased discharge from the *excretory vessels of the whole system.*

* Treatise on the Venereal, vol. ii. p. 272.

† Observations on the Venereal Disease, vol. i. p. 297.

To

To render plain truths still plainer, and to show, on this subject, more strikingly the incorrectness of Mr. Bell and of Mr. Howard, let it be recollected, that the effect of every preparation of mercury is uniformly evinced by a greater discharge of some of the secretions; and this effect is so constant, that it sometimes takes place from the use of the mineral in a crude state.* In short, as Mr. Hunter remarks, there is no proof of its affecting the constitution without this consequence; and its employment assuredly cannot be followed by any salutary result, unless it operates upon the constitution. But it is useless to dwell on this point, and there are, probably, few men of observation who will accede to the sentiment expressed by Mr. Bell. The opinion stated by Mr. Howard, of the necessity of profuse salivation in every case, had its advocates at a very early period in the history of this medicine; and though, perhaps, scarcely any opinion has, at different times, been more warmly embraced, or more indignantly rejected, it nevertheless, at the present day, is one of the most popular. It seems to owe this general reception to the well-known principles espoused by Mr. Hunter, that no two morbid actions can exist at the same time, and that one irritation destroys another. And yet nothing can be more evident, from Mr. Hunter's writings, than that this very practice met in him a decided enemy.

It is of minor consideration to be informed of the causes which have given origin to this mode of treatment; and painful indeed the recollection of the miseries it has created. No absurdity in medical practice has been the destruction of more lives; none the source of more pain and calamity. Well might Dr. Hoffman pronounce the abuse of this remedy, in the hands of the unskilful, to be more terrible than the sword. The pages of the older writers, as well as those of the modern, fully confirm the fact. Yet this method of cure is still *popular,* still pursued both in private practice and in public institutions.

From the theory which has already been given of the nature and character of the venereal, the constitution is the seat of the disease, and the indications of cure will accordingly be more readily fulfilled by the employment of those remedies which attack the disorder by their operation on the whole system. To obtain this end, mercury possesses superior claims, and those preparations of it which more directly act upon the secretory vessels of the surface, for the reasons before mentioned, are to be preferred. A pre-

* Mead's Medical Works, vol. i, p. 106.

ternatural action from any one of the secretions is not to be depended on, as "it is only a proof of the susceptibility of some parts to such a stimulus,"* and the disease will remain uncured, as is shown from the stationary appearance of local affections.† By topical applications, too, local affections may assume the healthy character, and yet the constitution remain contaminated. Mercury, therefore, to employ the forcible language of Mr. Hunter, must be in a state of solution in the juices of the body.

The *murias hydrargyri*, or the corrosive sublimate, is recommended for these several purposes; a form of mercury which, like every other, has had many opponents and adherents. It is thought by some to have been first employed as an anti-venereal by Basil Valentine,‡ but, upon the authority of the celebrated Van Swieten, it came into general use only in 1754, and the favorable reports of its efficacy "would fill," says Mr. Pearson, "a volume of considerable magnitude. In the number of its advocates may be found the distinguished names of Locher, De Haen, Pringle, Cleghorn, Gorden, Russell, Stoll, and Lewis. Among the principal advantages which it possesses over that of every other preparation of the same remedy, are, that, judiciously administered, it is particularly mild and safe in its operation, will admit of a more extensive use in all the various forms of lues venerea, and subject the patient to fewer inconveniences: that it readily enters into the general circulation, becomes miscible with the several fluids of the body, the soonest arrests the progress of the complaint, and eliminates the morbid matter through those emunctories best calculated for that purpose: that it supersedes the necessity of salivation, by its action on all the secretions, and by promoting especially the cuticular discharges, and the evacuations from the kidneys: that it is the only preparation to be depended on in those peculiar habits of body so susceptible to become salivated by every other form of mercury now in use: that, in its ultimate effects upon the constitution, it is attended with comparatively no injury. These facts are truly important, and many of them are granted by those who altogether reject it.

It is not a little unfortunate for the advocates of the other forms of this medicine, that the objections which have been brought against the corrosive sublimate are so dissimilar?

* Hunter, ut antea, p. 344. See also Ferriar, vol. iii. p. 257.

† Hunter, p. 341. Saunders on the Liver, 4th ed. Appendix, p. 73.

‡ Pearson, ut antea, p. 100.

It

It has been assigned as a reason against the preparation itself, that it has failed of its salutary effects by being given in too small doses. By some its anti-venereal properties are said to be lost on account of its too readily exciting the cuticular discharge; by others it is owing to its defective action on the secretions of the skin and mouth. By some it is admitted to be beneficial in the primary stage of the disease, and by others it is contended that it is calculated to remove only secondary symptoms. It is also declared that it is violent and uncertain in its operation, and that it does not render the cure permanent.* Some of these objections are, indeed, weighty, and, were they well founded, would fully justify the abandonment of this peculiar combination of mercury; but, if the least reliance is to be placed upon the experience and observation of those who have employed the corrosive sublimate with the most disinterested and honorable views, and solely to determine upon its anti-venereal powers, evidence sufficient to prove their fallacy, derived from indubitable sources, might be adduced. The testimony of Dr. Locher, of the Vienna hospital, is so full and explicit, that it were an omission not to insert it. Having witnessed the "horrid calamities" arising from salivation and other abuses which existed in that institution in the management of venereal patients, upon the recommendation of Van Swieten, he made trial of the corrosive sublimate. From the year 1754 to 1762, he cured by it no less than 4880 persons, *without inducing salivation;* and testifies, that "no persons died, or experienced the least painful and dangerous symptoms, in consequence of this remedy."† In the cases in which the same preparation was recommended by Pringle,‡ the cures that were effected were permanent, and, from the repeated experience of many other writers, the same result ensued.

As some one or more of the articles of the vegetable kingdom are in general employed in those cases in which the corrosive sublimate is administered, it would not be irrelevant to examine how far they are entitled to particular confidence. It may not be improper to remark, that, of the many which have been or are now made use of, the *lignum guaiaci* and the *radix sarsaparillæ* seems to claim the first notice. They are acknowledged to be useful during the ad-

* Hunter, Howard, Pearson, Mathias, and others.

† Locher's Observat. Pract. as quoted by Van Swieten, Commentaries, vol. xvii. p. 294.

‡ Gorden's Lond. Med. Obs. and Inq. vol. i. p. 365. vol. ii. p. 73.

ministration of the muriate of mercury, in cases of recent affection; and in the secondary symptoms of the disease, for the evils which have taken place from the injudicious employment of mercury, &c. their salutary operation has been uniformly evinced.

Before concluding the present essay, it is but proper to state, that the preparation of mercury now recommended, has been employed for the last seventeen years in the private practice of Professor Hosack, and during his attendance at the New-York State Prison, New-York Hospital, and the Alms-house of this city, as physician of those institutions. It has invariably been found to be the remedy best calculated for the removal of lues venerea, both in its primary and secondary stages; and not a single case is recollected in which the cure has not been permanent. Those injurious effects upon the stomach and bowels, which are so much apprehended, were avoided by a cautious employment of the medicine, and by a due consideration of the peculiarities in the constitution and state of the patient. From this form of mercury, salivation scarcely ever was induced; and while under its influence, the employment of the decoct. guaiac. et sarsaparil. was found to be an excellent auxiliary in recent cases; and in the secondary stage of the disease, where the patient had been neglected, or when improprieties in the cure had been committed, it was almost indispensable.

To conclude. Though satisfied that the muriate of mercury possessed full claims to the title of a powerful anti-venereal, from a perusal of the testimony published in its favor, and from a personal knowledge of the result of several cases in which it had been employed; with the view of more fully determining so important a subject, and to ascertain as far as practicable whether the objections which have been stated against it, particularly those of the distinguished Mr. John Pearson, were founded in reality; at the suggestion of the writer, the use of the muriate of mercury was adopted, in the spring of the present year (1811), in the New-York Hospital, by Mr. John C. Cheesman, the house surgeon. From the extensive charity which this excellent institution affords, there was now abundant opportunity of seeing almost every form of this disease, from the more mild to the most aggravated; cases of recent infection and those of long standing. After a careful examination of the histories of a great variety of cases, a selection was made of several of those affected with the primary, and of others laboring under the secondary, stages of this disease. The corrosive sublimate was given, in some instances, in the form of the spirituous solution, and in other instances made into pills; the decoct. guaiac. et sarsaparil. was employed as an auxiliary, and occasional recourse was had to the application of the lunar caustic; but the external use of every preparation of mercury was omitted. The result of these several cases was attended with complete success.

